# Employee human resource management values: validation of a new concept and scale

**DOI:** 10.3389/fpsyg.2023.1049657

**Published:** 2023-05-05

**Authors:** Sophie Drouin-Rousseau, Claude Fernet, Stéphanie Austin, Bruno Fabi, Alexandre J. S. Morin

**Affiliations:** ^1^Département de Psychologie, Université du Québec à Trois-Rivières, Trois-Rivières, QC, Canada; ^2^Département de Gestion des Ressources Humaines, Université du Québec à Trois-Rivières, Trois-Rivières, QC, Canada; ^3^Substantive-Methodological Synergy Research Laboratory, Département de Psychologie, Université Concordia, Montreal, QC, Canada

**Keywords:** HRM values, HRM practices, measurement, exploratory structural equation modeling, scale validation

## Abstract

**Purpose:**

Although human resource management (HRM) practices all seek to support and improve organizational functioning, the value ascribed to various HRM practices differs greatly among employees. Drawing on an exhaustive measure of HRM practices, this study proposed a new conceptualization and measure of HRM values, the HRM Values Scale (HRM-VS).

**Design/methodology/approach:**

To examine the psychometric properties of scores obtained on this new measure, we rely on a sample of 979 employees occupying a variety of jobs within various private and public organizations.

**Findings:**

Through the comparison of confirmatory factor analysis (CFA) and exploratory structural equation modeling (ESEM) solutions, our results supported a nine-factor structure of participants’ responses to the HRM-VS and the measurement invariance of this solution across male and female employees. Specifically, they support that the HRM-VS items adequately capture core HRM values underlying independent HRM practices. Criterion-related validity was evidenced with respect to employees’ ratings of intrinsic and extrinsic job satisfaction.

**Research implications:**

The HRM-VS appears to represent a promising tool for research and intervention seeking to account for individual differences in the relative importance of various HRM practices, in order to devise more effective HRM systems.

**Practical implications:**

This new concise but complete measure could help better guide organizations in tailoring their strategic HRM.

**Originality/value:**

This study introduces HRM values as a valid concept that characterizes what employees desire or consider to be important in relation to HRM practices.

## 1. Introduction

Research in strategic human resource management (HRM) seeks to document the contribution of HRM systems, or of interrelated sets of HRM practices, to the performance and optimal functioning of employees and organizations (Combs et al., 2006). HRM practices include, for instance, selection, training and development, information sharing and communication, and human resource planning (Boon et al., 2019). Although previous research has generally demonstrated positive relations between these practices and various organizational outcomes (Jiang et al., 2012), organizational scholars have begun to express concerns about the variability of the effects of some types of HRM systems or practices on employees (Kuvaas and Dysvik, 2010; Han et al., 2020). For instance, research indicates that not all practices are able to equally support employee motivation (Jiang et al., 2012). As a result, questions arise as to whether the contribution of HRM practices is uniformly valued by, and beneficial for, all employees. In this regard, the research on work values suggests that the importance given to an object, such as HRM practices, could be decisive in helping us to understand how much value is ascribed to this object by individual employees. Unfortunately, inter-individual differences in the value ascribed to various HRM practices remain undocumented (Garg et al., 2021), which is worrisome given the colossal investments made by organizations to improve and support employee well-being and performance via HRM practices.

To address this issue, the present study seeks to broaden our understanding of HRM practices by offering a perspective focusing on the “relative importance” that employees place on individuals HRM practices, which we hereafter refer to as their HRM values. To achieve this objective, we first review how HRM practices have been conceptualized and operationalized in previous research in order to identify a core set of HRM practices. It is noteworthy that, despite growing empirical evidence regarding the importance of HRM systems and practices, a recent systematic review by Boon et al. (2019) suggests that more than 80% of the studies on the subject rely on measures that have never been properly validated. This underscores the need to carry out a thorough assessment of the psychometric properties of scores obtained on our proposed measure of HRM values, which is the objective of the present study. To achieve this objective, we rely on the items of a measure of HRM *practices* initially developed by Geringer et al. (2002), and revised by Fabi et al. (2015), as our starting point to examine HRM *values*. More precisely, rather than focusing on the presence or absence of each of these HRM practices as did Fabi et al. (2015), we examined the relative value employees attribute to diverse HRM practices using an alternative six-point response scale. To do so, we assess the *a priori* factor structure of employees’ ratings of the importance they attribute (HRM values) to each of the HRM practices included in Fabi et al.’s (2015) measure, before introducing an improved shorter version of this measure, the HRM value scale (HRM-VS). We finally investigate the factor validity, reliability, measurement invariance across subsamples of male and female employees, and criterion-related validity (in relation to measures of intrinsic and extrinsic job satisfaction) of scores obtained on this measure.

Drawing on the research literature on work values (e.g., Super, 1980; Ros et al., 1999), we broadly define work values as beliefs about desirable behaviors (e.g., working with people) or end-states (e.g., social recognition, accomplishment) at work. These values are guiding principles for evaluating work outcomes (e.g., job satisfaction) or settings (e.g., job characteristics) and for choosing among different work alternatives (Ros et al., 1999). Typically assessed by the importance that people attribute to different facets of their work environments (Busque-Carrier et al., 2022), work values are associated with a variety of work-related attitudes, behaviors, social interactions, and roles (Ros et al., 1999). Work values refer to what employees value and seek to obtain out of their work lives in general, such as status (e.g., being part of a prestigious organization), intrinsic (e.g., being able to use creativity), extrinsic gains (e.g., to have a good salary), and social relationships (e.g., having an understanding boss) (Busque-Carrier et al., 2022). HRM values are more specific and refer to the set of HRM practices that employees purposely seek from an employer (e.g., fair wages, job flexibility, training programs). Thus, whereas work values cover a generic range of elements employees seek to obtain at work, HRM values form a subset of these work values that are specific to the type of HRM practices employees would like to see implemented in their workplace. As a result, HRM values should be critical in understanding the effectiveness of a HRM system, as well as inter-individual differences in the relative role played by each HRM practice for specific employees. Failure to consider these HRM values (i.e., employees’ opinion about the worth and importance of specific HRM practices), would thus make it difficult to identify a core set of HRM practices that would be beneficial to the organization and to most of its employees. As a result, taking HRM values into account may be as important as documenting the relative efficacy of the HRM practices themselves, as these values are likely to play a central role in influencing this efficacy.

Arguably, the most comprehensive measure of HRM practices available to date has been developed by Geringer et al. (2002). Based on an extensive literature review and an international committee of HRM experts, these authors developed a measure that focuses on six distinct sets of HRM practices (i.e., selection, training and development, performance appraisal, compensation, leadership and communication). Unfortunately, whereas this measure has been used across a variety of linguistic versions in more than 40 different countries, its psychometric properties remain unknown. More recently, a French-Canadian version of this measure was proposed by Fabi et al. (2015). In addition to their linguistic adaptation, Fabi et al. (2015) also incorporated four additional sets of HRM practices based on their more recent comprehensive review of the literature: work-life balance, induction, benefits, and work design (although the authors never reported the results associated with this last dimension). The resulting measure thus incorporates 73 items, assessing 10 distinct HRM practices; selection (i.e., identification of recruitment needs, selection and attraction procedures; Robbins et al., 2015), training and development (i.e., acquiring the knowledge, skills or abilities necessary to perform work duties; Robbins et al., 2015), induction (i.e., employee integration to their new job, colleagues and the work environment; Kelley, 2004), leadership (i.e., behavior and role of the supervisor or immediate superior; Robbins et al., 2015), performance appraisal (i.e., measuring employee performance against pre-established objectives; Werther and Davis, 1996; Robbins et al., 2015), compensation (i.e., system based on the nature of the job and the work environment, which determines workers’ base salary and bonuses; Armstrong and Taylor, 2014), benefits (i.e., financial and social advantages offered to employees; Armstrong and Taylor, 2014), communication and participation (i.e., circulation and sharing of information and participation in decision-making; Wagner, 1994; Uhl-Bien et al., 2018), work-life balance (i.e., practices designed to create a healthy equilibrium between work and personal life; Robbins et al., 2015), and work design (i.e., practices seeking to improve work design, such as empowerment; Parker et al., 2017). The resulting list of HRM practices is entirely aligned with systematic reviews of the most relevant types of HRM practices to consider in research and practice (Lepak et al., 2006; Boon et al., 2019). Importantly, Boon et al. (2019) noted that six types of practices seemed particularly important: training and development, participation, compensation, performance appraisal, selection, and job characteristics. They further suggest to not overlook any relevant HRM practices likely to impact HRM systems, such as benefits, leadership-supervision, or work-life balance (also see Chuang and Liao, 2010).

As part of their data collection, Fabi et al. (2015) relied on two different rating scales. The first, which is the only one used in the analyses reported in their original study, was designed to assess the presence or absence of each practice using a yes-no format. In contrast, the second rating scale asked employees to indicate how much importance (value) they attributed to each HRM practice using a six-point rating scale ranging from “very low importance” to “very high importance”. This response format is commonly used in research on work values (Ros et al., 1999; Busque-Carrier et al., 2022), and provides a way to directly assess the value attributed to each practice by the employees. In the present study, we rely on the data initially collected, but never analyzed, by Fabi et al. (2015) to investigate the psychometric properties of employees’ ratings of HRM values obtained using this second response scale. In addition, as the length of the original instrument represents a considerable challenge to its widespread utilization in research and practice, we also propose a shorter version of this measure (including four items per subscale), adapted to the assessment of HRM values.

The present study seeks to contribute to research on HRM practices by proposing a concise but comprehensive measure of HRM values, allowing us to more systematically investigate the importance ascribed by individual employees to a series of HRM practices. More precisely, using a combination of Confirmatory Factor Analyses (CFA) and Exploratory Structural Equation Modeling (ESEM; Asparouhov and Muthén, 2009; Morin et al., 2013), we first investigated the factor validity and composite reliability of a measure initially developed by Geringer et al. (2002) and Fabi et al. (2015), adapted to the assessment of HRM values. We then propose a shorter version of this instrument including four-item subscales (corresponding approximately to a 50% reduction of the number of items). The items retained in this short version were selected based on the results from our analyses (i.e., factor loadings, cross-loadings, modification indices and covariance residuals) and the consultation of a panel of five experts in the field following current recommendations for short-scale development (Smith et al., 2000; Marsh et al., 2005; Maïano et al., 2008; Perreira et al., 2018). We also investigate the factor validity, scale sore reliability, composite reliability, measurement invariance across subsamples of male and female employees, and criterion-related validity (in relation to ratings of intrinsic and extrinsic job satisfaction; Weiss et al., 1967) of scores obtained on this new instrument. For this last step, the decision to rely on criterion measures of intrinsic and extrinsic job satisfaction is underpinned by self-determination theory (Ryan and Deci, 2017), which led us to expect that HRM values related to selection, training and development, induction, communication and participation, work-life balance and work design should be positively associated with higher levels of intrinsic job satisfaction, whereas HRM values related to compensation, benefits, performance appraisal and leadership should be positively associated with higher levels of extrinsic job satisfaction. Indeed, according to self-determination theory (Ryan and Deci, 2017), HRM practices geared toward the reliance on external forms of motivators (such as compensation, benefits, performance appraisal and leadership) should be more strongly associated with extrinsic motives (doing something for instrumental reasons; e.g., avoiding constraints or obtaining material or social rewards) than to intrinsic motives (doing something for its own sake; e.g., enjoyment, interest).

## 2. Methods

### 2.1. Participants and procedure

The data used in this study comes from Fabi et al. (2015) study of HRM practices in Canadian organizations recruited from a university-based center. Their study was part of a larger project on HRM practices and employee retention. The sample included 979 employees (51.3% women) with an average age of 33.5 years (SD = 11.46) and tenure of 4.2 years (SD = 4.2). About one third of participants worked in the manufacturing industry (31.2%), in the service industry (31.3%) and in the public sector (35.3%). Although no incentives were offered to participants, they were authorized by their organization to respond to a 30-min survey during working hours. In return to participation, the organizations were provided with a customized report on their capacity to retain their employees. Approval was obtained from the original authors’ university research ethics committee.

### 2.2. Measures

#### 2.2.1. HRM values

Participants completed Fabi et al.’s (2015) adapted French-Canadian version of the Geringer et al.’s (2002) scale assessing 10 distinct HRM practices: (1) Selection (8 items; e.g., *Work experience in a similar job*); (2) Induction [2 items; e.g., *Organization of social activities* (*sports, outdoor activities, holidays,* etc.)]; (3) Training and development (9 items; e.g., *Improving technical abilities of employees*); (4) Leadership (8 items; e.g., *Treats me with respect*); (5) Performance appraisal (8 items; e.g., *Helping the employee in improving his performance*); (6) Compensation (7 items; e.g., *Part of the remuneration is based on the knowledge and skills of employees*); (7) Benefits (8 items; e.g., *Collective insurance*); (8) Communication and participation (7 items; e.g., *Possibility to take decisions related to my job*); (9) Work-life balance (6 items; e.g., *Having the opportunity to take long-term leave*); (10) Work design (10 items; e.g., *Being free to explore my own ideas*). Participants were asked to indicate on a 6-point scale ranging from 1 (*very low importance*) to 6 (*very high importance*) the degree of importance they ascribed to each HRM practice.

#### 2.2.2. Job satisfaction

A 18-item version of the Minnesota Satisfaction Questionnaire by Weiss et al. (1967); French version by Mathieu et al., 2016 was used to assess intrinsic (12 items; e.g., *The feeling of having accomplished something through my job*) and extrinsic job satisfaction (6 items; *My opportunities of advancement in this job*). Participants indicated how they felt about each statement on a 6 point-scale ranging from 1 (*very weak satisfaction*) to 6 (*very strong satisfaction*). Previous studies support the scale score reliability and validity of these scales (e.g., Weiss et al., 1967; Fabi et al., 2015).

### 2.3. Analyses

All analyses were conducted with Mplus 7.4 (Muthén and Muthén, 2015) using the Maximum Likelihood Robust (MLR) estimator, which is robust to non-normality (Finney and DiStefano, 2006). The few missing responses (1.57%) were handled with Mplus default procedure of Full Information Maximum Likelihood (FIML; Enders, 2010). Analyses were conducted in two steps: (1) an in-depth examination of the full version (measurement models: goodness-of-fit indices, parameter estimates, factor correlations and reliability) and (2) an in-depth validation of a short version (measurement models, reliability, measurement invariance across sex, and criterion-related validity).

### 2.4. Measurement models

The factor structure of the original (i.e., 73 items) version of the HRM-VS was investigated by comparing CFA and ESEM representations. In both representations, the *a priori* 10 correlated factors were defined by their *a priori* indicators. Whereas the CFA representation constrained all cross-loadings between the items and the non-target factors to be exactly zero, the ESEM solution allowed for all of these cross-loadings to be freely estimated, but *targeted* to be as close to zero as possible using a confirmatory factor rotation procedure (i.e., target rotation; Asparouhov and Muthén, 2009; Morin et al., 2020). Statistical research generally indicates that the free estimation of cross-loadings tends to result in more appropriate factor definition and more accurate estimates of factor correlations when cross-loadings as small as 0.10 are present in the population model, but to remain unbiased even when no cross-loadings are truly present in the data (for a review, see Asparouhov et al., 2015). These two solutions were compared using goodness-of-fit indices (Marsh et al., 2005): The Comparative Fit Index (CFI), the Tucker-Lewis index (TLI) and the Root Mean Square Error of Approximation (RMSEA) with its confidence interval. According to prevailing guidelines, CFI and TLI values ≥0.90 and RMSEA values ≤0.08 indicate an acceptable level of model fit, whereas CFI and TLI values ≥0.95 and RMSEA values ≤0.06 indicate an excellent level of model fit. Although we also report the MLR chi-square (*χ*^2^), this additional indicator will not be considered due to its known over sensitivity to sample size and minor misspecifications (Marsh et al., 2005). Model selection will also be guided by an examination of the parameter estimates (factor loadings, cross-loadings, and correlations), where the presence of similarly well-defined factors accompanied by lower factor correlations can be taken as evidence for the superiority of the ESEM solution (Morin et al., 2020).

“The parameter estimates from these two solutions will also inspected to select the subset of items to be retained for the development of the short version of the HRM-VS”. This selection of item followed current published state-of-the-art guidelines for the development of questionnaire short-forms (Smith et al., 2000; Marsh et al., 2005; Maïano et al., 2008; Perreira et al., 2018). Thus, items associated with stronger factor loadings (minimally ≥0.30, but ideally ≥0.400; Morin et al., 2020), small cross-loadings (minimally ≤0.300 but ideally ≤0.200; Morin et al., 2020), small modification indices, and small covariance residuals will be favored for inclusion into the final version. All items that seemed appropriate based on these methodological criteria were then submitted to a panel of five experts asked to determine the items that best represent HRM values underlying key HRM practices across a variety of organizational settings, and covering non-redundant facets of these practices. In this second comparison, the goal was to retain the four most suitable items for each subscale, while making sure to retain an adequate coverage of the content of each scale (Nunnally and Bernstein, 1994). In this regard, the target number of 4 items per scale corresponds roughly to a reduction of 50% in the length of the questionnaire, a ratio found to be suitable in previous research on short-form development (e.g., Marsh et al., 2005; Morin et al., 2016). The final set of items was then analyzed through the same CFA versus ESEM comparison used for the longer version of the scale.

### 2.5. Reliability

The parameter estimates from the retained measurement model were used to assess the composite reliability of scores on each of the resulting factors. Composite reliability estimates were obtained via the calculation of McDonald’s (1970) omega coefficient (*ω*), which directly considers the relative contribution of each item (i.e., the factor loadings, reflecting reliable variance) to the definition of the factors and the item specific uniquenesses (including random measurement error) (e.g., Sijtsma, 2009; Morin et al., 2020). We also report H as an indicator of construct reliability calculated from the standardized factor loadings (Hancock and Mueller, 2001; Rodriguez et al., 2016; Morin et al., 2020).

### 2.6. Measurement invariance

Evidence of measurement invariance is of outmost importance to assess the ability to generalize, and compare, the results obtained using a measurement instrument to members of different groups. In the present study, we investigate the generalizability (i.e., measurement invariance) of ratings obtained on the final version of the HRM-VS across samples of male and female participants. These tests were realized in the following sequence (Millsap, 2011): (a) Configural invariance (same measurement model with no added constraints); (b) weak invariance (invariance of the factor loadings); (c) strong invariance (invariance of loadings and intercepts); (d) strict invariance (invariance of loadings, intercepts and uniquenesses); (e) invariance of the latent variances-covariances (invariance of loadings, intercepts, uniquenesses and latent variances-covariances); (f) latent means invariance (invariance of loadings, intercepts, uniquenesses, latent variances-covariances and latent means). In these tests, a decrease in CFI and TLI values greater than 0.10, and an increase in RMSEA values of 0.015 or more between a model and the previous one in the sequence suggests that the invariance hypothesis should be rejected (Marsh et al., 2005; Chen, 2007).

### 2.7. Criterion-related validity

Tests of criterion-related validity were finally conducted by incorporating latent CFA factors representing intrinsic and extrinsic job satisfaction as outcomes to the final retained measurement model (i.e., these outcomes factors were predicted by the HRM-VS factors).

## 3. Results

### 3.1. Factor structure of the complete HRM-VS

The CFA solution estimated on the original version of the HRM-VS resulted in an unacceptable level of fit to the data according to the CFI and TLI (*χ*^2^ = 6958.800; df = 2,510; CFI = 0.799; TLI = 0.789; RMSEA = 0.043; with a 90% confidence interval of 0.041 to 0.044). However, most factors were well-defined by most of their items in this solution, with factor loadings ranging from 0.170 to 0.810 (*M* = 0.601). The exact parameter estimates from this solution are reported in Supplementary Table S1 (factor loadings and uniquenesses) and Supplementary Table S2 (factor correlations) of the online supplements. Although the ESEM solution provided a better fit to the data than its CFA counterpart (*χ*^2^ = 4238.885; df = 1,943; CFI = 0.896; TLI = 0.856; RMSEA = 0.035; confidence interval = 0.033 to 0.036), it remained unsatisfactory according to the CFI and TLI. The exact parameter estimates from this solution are reported in Supplementary Table S3 (factor loadings and uniquenesses) and Supplementary Table S2 (factor correlations) of the online supplements. An examination of these parameter estimates indicate that the factors appeared to be more weakly defined in the ESEM solution than in the CFA solution, with target loadings ranging from 0.037 to 0.887 (*M* = 0.483). More precisely, more than half of the factors appeared to be weakly defined by a majority of their items: Leadership (*λ* = 0.166 to 0.837; *M* = 0.494), selection (*λ* = 0.220 to 0.532; *M* = 0.381), compensation (*λ* = 0.124 to 0.745; *M* = 0.433), benefits (*λ* = 0.089 to 0.819; *M* = 0.556), and work design (*λ* = 0.117 to 0.627; *M* = 0.390). In addition, with only two indicators, the induction factor appeared to be particularly problematic (*λ* = 0.037 and 0.042; *M* = 0.040). We decided not to retain this factor for the development of the short version. In addition, the ESEM solution also revealed some cross-loadings (|*λ*| = 0 to 0.433; *M* = 0.059). However, only 18 of those (out of 657) were higher than 0.200. Of those, 10 items had strong cross-loading but retained their main loading on their *a priori* factor, whereas the remaining 8 items (two of which were associated with the problematic induction factor) had their highest loading on a non-target factor. This second set of items were automatically excluded from the short version. Finally, and supporting the value of this representation, factor correlations were much smaller in the ESEM solution (*r* = 0.012 to 0.478; *M* = 0.245) than in the CFA solution (*r* = 0.231 to 0.728; *M* = 0.506).

### 3.2. Factor structure of the short HRM-VS

Following a detailed examination of the parameter estimates from the CFA and ESEM solution estimated on the complete version of the HRM-VS and the consultation of our panel of expert, a short version of the HRM-VS was constructed. In this version, a total of 36 items are used to define 9 correlated factors (selection, training and development, leadership, performance appraisal, compensation, benefits, communication and participation, work-life balance, and work design), with four items associated with each factor. As for the long version, the CFA solution estimated on this shorter version of the HRM-VS failed to achieve a satisfactory level of fit to the data (*χ*^2^ = 1504.587; df = 558; CFI = 0.903; TLI = 0.890; RMSEA = 0.042; confidence interval = 0.039 to 0.044) according to the TLI. In contrast, the ESEM solution resulted in a fully acceptable level of fit to the data (*χ*^2^ = 807.498; df = 342; CFI = 0.952; TLI = 0.912; RMSEA = 0.037; confidence interval = 0.034 to 0.041). Parameter estimates associated with these solutions are reported in Table 1 (factor loadings and uniquenesses) and Table 2 (CFA and ESEM factor correlations).

**Table 1:** Standardized factor loadings (*λ*) and item uniquenesses (*δ*) for the HRM-VS measurement models.

Items	CFA	ESEM	*λ*	*δ*	F1 (*λ*)	F2 (*λ*)	F3 (*λ*)	F4 (*λ*)	F5 (*λ*)	F6 (*λ*)	F7 (*λ*)	F8 (*λ*)	F9 (*λ*)	*δ*
F1: Work-life balance												
B1AA	0.600**	0.604**	0.550**	0.001	−0.029	0.044	−0.072	0.008	−0.023	−0.034	0.153*	0.665**
B1BB	0.322**	0.896**	0.400**	0.143*	−0.080	0.003	0.033	0.073	−0.006	−0.140*	−0.050	0.822**
B1DD	0.615**	0.621**	0.558**	−0.082	0.046	0.025	−0.014	−0.065	0.081	0.003	0.064	0.646**
B1FF	0.431**	0.814**	0.401**	0.052	0.004	−0.010	−0.018	0.016	0.005	0.192**	−0.116*	0.766**
F2: Leadership												
B2CC	0.700**	0.510**	0.012	0.636**	−0.012	−0.042	0.019	0.068	−0.026	0.015	0.141**	0.492**
B2DD	0.789**	0.377**	−0.005	0.858**	−0.004	0.009	−0.047	−0.066*	0.004	0.059*	0.034	0.277**
B2EE	0.677**	0.542**	0.036	0.435**	0.104	0.028	0.142*	0.057	−0.051	−0.031	−0.072	0.555**
B2FF	0.553**	0.694**	0.062	0.507**	0.135*	0.027	0.078	0.003	0.112*	−0.016	−0.036	0.691**
F3: Communication and participation												
B3BB	0.636**	0.595**	0.009	0.179*	0.401**	0.204**	−0.026	0.046	0.016	0.034	−0.093*	0.597**
B3DD	0.601**	0.639**	−0.043	0.021	0.424**	0.142*	0.020	0.198**	−0.022	0.016	−0.071	0.633**
B3EE	0.718**	0.485**	0.002	−0.009	0.822**	−0.074	0.011	−0.001	0.019	0.018	−0.007	0.373**
B3FF	0.680**	0.538**	0.001	0.006	0.630**	0.009	−0.017	−0.079*	0.007	−0.014	0.233**	0.462**
F4: Performance appraisal												
B4CC	0.726**	0.472**	−0.004	0.152*	0.078	0.583**	−0.022	−0.041	0.045	0.029	0.065	0.462**
B4DD	0.845**	0.286**	0.057	−0.036	0.038	0.795**	0.022	0.062*	−0.048*	−0.001	0.031	0.287**
B4EE	0.813**	0.338**	0.025	−0.021	0.048	0.796**	0.035	0.010	0.040	−0.009	−0.064*	0.318**
B4FF	0.807**	0.349**	−0.017	−0.045	−0.003	0.827**	0.015	−0.020	0.020	0.034	0.021	0.323**
F5: Selection												
B5CC	0.508**	0.742**	0.001	0.050	0.042	0.038	0.389**	0.074	−0.041	−0.058	0.122*	0.756**
B5DD	0.630**	0.604**	−0.045	0.017	−0.005	0.008	0.608**	0.101*	−0.005	0.014	−0.041	0.583**
B5EE	0.534**	0.715**	0.011	0.053	−0.106*	0.048	0.488**	−0.007	0.044	0.122**	−0.012	0.697**
B5FF	0.530**	0.719**	−0.023	0.018	0.075	−0.015	0.537**	−0.111*	0.059	−0.005	0.084*	0.676**
F6: Training and development												
B7CC	0.648**	0.580**	0.003	0.011	−0.027	0.187**	0.146*	0.414**	0.123*	−0.031	0.080	0.558**
B7EE	0.682**	0.534**	0.011	0.065	0.044	0.005	−0.089*	0.698**	0.017	0.027	−0.057	0.482**
B7FF	0.755**	0.430**	−0.004	−0.049	0.061	−0.095	0.001	0.824**	0.018	0.027	0.014	0.338**
B7HH	0.691**	0.523**	0.018	−0.008	−0.010	0.014	0.058	0.558**	−0.029	0.044	0.166**	0.540**
F7: Compensation												
B8AA	0.561**	0.685**	0.109	0.018	0.091	−0.141*	0.087	0.052	0.527**	0.093*	−0.102*	0.624**
B8BB	0.652**	0.574**	0.114	−0.069	0.007	−0.013	0.067	−0.010	0.668**	−0.016	−0.127*	0.515**
B8DD	0.781**	0.391**	−0.084	0.051	−0.028	0.034	−0.062	−0.035	0.839**	−0.088*	0.080	0.316**
B8EE	0.689**	0.525**	−0.055	−0.008	−0.057	0.099*	−0.036	0.083	0.619**	0.055	0.132*	0.512**
F8: Benefits												
B9BB	0.762**	0.419**	−0.077	0.053	−0.027	0.014	0.074	0.003	0.023	0.733**	0.006	0.423**
B9CC	0.733**	0.463**	0.007	0.081*	0.003	0.070*	−0.054	0.066	−0.024	0.647**	0.036	0.476**
B9GG	0.815**	0.336**	−0.024	−0.032	0.059	−0.034	−0.062	−0.013	0.063*	0.855**	0.035	0.277**
B9HH	0.712**	0.493**	0.134*	−0.055	−0.005	0.004	0.110	0.013	0.075	0.664**	0.017	0.453**
F9: Work design												
B10AA	0.610**	0.628**	0.033	−0.072	0.080	0.038	0.126*	−0.009	−0.022	0.094*	0.531**	0.582**
B10BB	0.753**	0.433**	0.057	0.023	0.138	0.002	0.056	0.014	0.022	0.011	0.670**	0.387**
B10CC	0.782**	0.388**	0.028	0.126*	0.023	0.031	0.012	0.136*	0.021	0.022	0.588**	0.416**
B10DD	0.735**	0.459**	0.031	0.144*	−0.013	0.043	−0.037	0.099*	0.063	0.073	0.561**	0.460**

*p < 0.05; **p < 0.01; CFA, Confirmatory factor analysis; ESEM, Exploratory structural equation modeling; B1, Work-life balance items; B2, Leadership items; B3, Communication and participation items; B4, Performance appraisal items; B5, Selection items; B7, Training and development items; B8, Compensation items; B9, Benefits items; B10, Work design items; *λ* = standardized factor loading (main factor loadings are in bold); δ = standardized item uniquenesses.

**Table 2:** Factor correlations from the confirmatory factor analytic (CFA; under the diagonal) and exploratory structural equation modeling (ESEM; above the diagonal) solution of the HRM-VS.

	F1	F2	F3	F4	F5	F6	F7	F8	F9
F1	–	0.199*	0.229**	0.170*	0.139*	0.173*	0.307**	0.269**	0.199*
F2	0.280**	–	0.508**	0.425**	0.231**	0.442**	0.216**	0.271**	0.336**
F3	0.290**	0.665**	–	0.526**	0.318**	0.401**	0.209**	0.339**	0.462**
F4	0.255**	0.505**	0.649**	–	0.337**	0.417**	0.318**	0.201**	0.340**
F5	0.166*	0.392**	0.443**	0.451**	–	0.360**	0.278**	0.331**	0.224**
F6	0.244**	0.537**	0.555**	0.511**	0.507**	–	0.249**	0.429**	0.375**
F7	0.341**	0.289**	0.264**	0.372**	0.335**	0.341**	–	0.121*	0.216**
F8	0.302**	0.376**	0.431**	0.289**	0.428**	0.522**	0.154*	–	0.308**
F9	0.363**	0.574**	0.667**	0.513**	0.444**	0.623**	0.350**	0.489**	**–**

*p < 0.05; **p < 0.01; F1, Work-life balance; F2, Leadership; F3, Communication and participation; F4, Performance appraisal; F5, Selection; F6, Training and development; F7, Compensation; F8, Benefits; F9, Work design.

These results reveal similarly well-defined factors for both the CFA (*λ* = 0.322 to 0.845; *M* = 0.668) and ESEM (*λ* = 0.400 to 0.855; *M* = 0.612) solutions. In addition, although cross-loadings were present in the ESEM solution, they remain generally small (|*λ*| = 0 to 0.233; *M =* 0.019), smaller than the target loadings, and only 2 of them (out of 288) were higher than 0.200, while 35 were between 0.100 and 0.200. Finally, factor correlations where once again smaller in the ESEM (*r* = 0.121 to 0.526; *M* = 0.302) solution than in the CFA solution (*r* = 0.154 to 0.665; *M* = 0.414), lending further support to the superiority of the ESEM solution, which was retained for further analyses. In this solution, most factors had an acceptable to satisfactory level of composite and construct reliability (except the work-life balance and selection factors which had suboptimal reliability coefficients): (1) work-life balance *ω* = 0.556 and H = 0.559; (2) leadership *ω* = 0.746 and H = 0.801; (3) communication and participation *ω* = 0.715 and H = 0.759; (4) performance appraisal *ω* = 0.866 and H = 0.860; (5) selection *ω* = 0.601 and H = 0.597; (6) training and development *ω* = 0.764 and H = 0.788; (7) compensation *ω* = 0.781 and H = 0.807; (8) benefits *ω* = 0.837 and H = 0.843; (9) work design *ω* = 0.749 and H = 0.687.

### 3.3. Measurement invariance

The results from the tests of measurement invariance conducted on the retained ESEM solution of the short HRM-VS are reported in Table 3. Although the TLI appears to be unsatisfactory for the initial model of configural invariance, this lack of fit seems to reflect the lack of parsimony of this solution, which involved the free estimation of all parameters across groups. Supporting this assertion, the TLI (as well as all other indices) are fully satisfactory for all other steps of the measurement invariance sequence. More importantly, none of the added invariance constraints resulted in a sufficient decrease in model fit to justify rejecting the invariance hypothesis. Thus, these results seem to support the complete measurement invariance of the HRM-VS ESEM measurement model across samples of male and female employees.

**Table 3:** Measurement invariance across sex for the HRM-VS.

Model	MLR *χ*^2^	df	CFI	TLI	RMSEA	90% CI RMSEA	∆χ*^2^* (*df*)	∆CFI	∆TLI	∆RMSEA
Configural invariance	1,289.761*	684	0.939	0.888	0.043	[0.039; 0.046]	–	–	–	–
Weak invariance	1,490.855*	927	0.944	0.923	0.035	[0.032; 0.039]	257.841 (243)	+0.005	+0.035	−0.008
Strong invariance	1,577.013*	954	0.938	0.918	0.037	[0.033; 0.040]	101.498 (27)	−0.006	−0.005	+0.002
Strict invariance	1,642.672*	990	0.935	0.917	0.037	[0.034; 0.040]	63.546 (36)	+0.001	−0.001	0.000
Var.-Covar. invariance	1,726.632*	1,035	0.931	0.916	0.037	[0.034; 0.040]	8.630 (45)	0.000	−0.001	0.000
Latent means invariance	1,785.508*	1,044	0.926	0.911	0.038	[0.035; 0.041]	5.537 (9)	−0.005	−0.005	+0.001

*p < 0.01; MLR *χ*^2^, robust chi-square test of exact fit; df, degrees of freedom; CFI, Comparative Fit Index; TLI, Tucker-Lewis index; RMSEA, Root Mean Square Error of Approximation; CI, RMSEA 90% Confidence Interval; ∆*χ*^2^, chi-square difference tests.

### 3.4. Criterion-related validity

Tests of criterion-related validity were realized by incorporating latent factors representing intrinsic and extrinsic job satisfaction to the final ESEM solution. The measurement model including all of these latent factors resulted in an excellent level of fit to the data (intrinsic satisfaction: *χ*^2^ = 1928.935; df = 1,062; CFI = 0.938; TLI = 0.934; RMSEA = 0.029; confidence interval = 0.027 to 0.031; extrinsic satisfaction: *χ*^2^ = 1213.720; df = 801; CFI = 0.965; TLI = 0.963; RMSEA = 0.023; confidence interval = 0.020 to 0.026), and revealed that both additional factors were also associated with satisfactory estimates of composite reliability (intrinsic satisfaction *ω* = 0.871; extrinsic satisfaction *ω* = 0.815). Interested readers can consult the latent correlations estimated as part of this model in Supplementary Table S4 of the online supplements. However, for purposes of assessing the criterion-related validity of scores obtained on the various HRM-VS factors, this model was converted to a predictive model to allow scores on the HRM-VS factors to predict scores on the intrinsic and extrinsic job satisfaction factors. The results from these predictive analyses are reported in Table 4. These results show that HRM values toward performance appraisal, communication and participation, and work design were positively associated with intrinsic job satisfaction, whereas leadership was negatively associated with this outcome. HRM values toward leadership, performance appraisal and selection were also positively associated with extrinsic job satisfaction, whereas communication and participation and benefits were negatively associated with this outcome. No significant relations were found for the other HRM values (training and development, compensation, and work-life balance).

**Table 4:** Criterion-related validity between HRM values and outcomes (intrinsic and extrinsic job satisfaction) for the HRM-VS.

	Intrinsic job satisfaction			Extrinsic job satisfaction		
	*b*	s.e.	*β*	*b*	s.e.	*β*
Work-life balance	−0.047	0.028	−0.089	−0.122	0.068	−0.100
Leadership	−0.057*	0.029	−0.106*	0.224*	0.072	0.184*
Communication and participation	0.081*	0.038	0.151*	−0.214*	0.087	−0.176*
Performance appraisal	0.109**	0.031	0.206**	0.190*	0.071	0.157*
Selection	0.039	0.027	0.074	0.228**	0.065	0.188**
Training and development	−0.034	0.027	−0.064	−0.003	0.064	−0.002
Compensation	−0.030	0.024	−0.056	−0.070	0.059	−0.057
Benefits	−0.009	0.026	−0.017	−0.125*	0.055	−0.103*
Work design	0.102**	0.029	0.192**	0.075	0.061	0.062

*p < 0.05; **p < 0.01; b, Unstandardized regression coefficients; s.e., Standard error; *β*, Standardized regression coefficient.

## 4. Discussion

This study was designed to validate a short and yet comprehensive measure of employees’ HRM values built from a measure initially developed by Geringer et al. (2002) and adapted by Fabi et al. (2015) to assess HRM practices. Using a combination of CFA and ESEM model, we were able to identify a set of 36 items, allowing us to assess the relative importance, or value, ascribed by employees to nine distinct HRM practices: Selection, training and development, leadership, performance appraisal, compensation, benefits, communication and participation, work-life balance, and work design. In addition, our results supported the complete equivalence (i.e., measurement invariance) of scores obtained on our measure across samples of male and female employees, making it suitable for research seeking to better understand how to maximize female integration in traditionally male-dominated work environments, as well as for organizations seeking to achieve a greater level of gender equality in term HRM practices (e.g., Rowe and Snizek, 1995).

In relation to the criterion-related validity of scores obtained on the HRM-VS, our results supported the presence of the expected positive associations between HRM values toward communication and participation and work design, and employees’ levels of intrinsic job satisfaction. These results are consistent with self-determination theory (Ryan and Deci, 2017), which highlights the role of practices that help to maximize the fit between employees own needs and values and their work environments, as well as with the results from previous research in which similar associations have been reported (e.g., Guest, 2002; Cartwright and Holmes, 2006; Brown et al., 2008). In contrast, leadership values were negatively associated with intrinsic job satisfaction and positively associated with extrinsic job satisfaction. One possible explanation for this result could be that employees’ may have interpreted the leadership items as reflecting a subtle attempt to gain control or manipulate them rather than as a genuine form of support. Although they might see this as important to the proper execution of the job, they may also not like it intrinsically.

Also contrary to our predictions, HRM values toward selection and communication and participation were positively associated with extrinsic job satisfaction, whereas benefits was associated negatively with this outcome. Furthermore, HRM values toward performance appraisal was associated with extrinsic job satisfaction, as expected, but was also found to be associated with higher levels of intrinsic job satisfaction. As suggested by Poon (2004), the way employees perceive performance appraisal might affect how it impacts their job satisfaction. Thus, when employees perceive performance appraisal as a punishment or form of control, this practice becomes more likely to negatively impact their intrinsic job satisfaction. In contrast, when performance appraisal is seen as a way to get constructive performance feedback, then it should help increase intrinsic job satisfaction. Arguably, similar perceptual differences might contribute to explain the inconsistent results found in relation to communication and participation (which could be seen as a source of genuine or instrumental recognition), selection (which could be seen as a way to maximize person-environment fit, as well as a way to exclude employees who do not fit a pre-established mold) and benefits (which can be seen as fair and equitable or not). These hypotheses need to be verified in future research.

Finally, HRM values toward some practices (training and development, compensation and work-life balance) did not show any association with either extrinsic or intrinsic job satisfaction. Because these HRM practices have been consistently associated with employees’ job satisfaction (Pendleton and Poutsma, 2004; Garcia, 2005; Morris et al., 2006; Brown et al., 2008; Petrescu and Simmons, 2008; Den Hartog et al., 2013), a question remains open regarding whether HRM values might be associated with a different set of outcomes than those typically found to be associated with their corresponding HRM practices. One promising research avenue would be to delve further into how HRM values coexist within individuals, and how individuals presenting these distinct HRM value profiles react to a variety of HRM practices.

### 4.1. Theoretical and methodological implications

Arguably, our core contribution lies in introducing HRM values as a valid concept that characterize what employees desire or consider to be important in relation to HRM practices. These values may reflect individual differences in how employees perceive, interpret, and react to the HRM practices prevailing, or lacking, in their work settings. By focusing on these HRM values, we depart from the traditional view that HRM practices will necessarily influence every employee in the same manner to HRM practices, to rather highlight the fact that each employee is likely to react differently to distinct HRM practices based on what they consider important to them. In doing so, this study adds to a new stream of research seeking to better understand the sources of the known variability in employees’ reactions to HRM practices. Whereas a previous study (Garg et al., 2021) showed that the salience of HRM practices explained variation in employee outcomes, our findings bring unique information about the relative value employees attribute to HRM practices. Additional research is needed to more precisely investigate whether and how these HRM values interact with HRM practices or other individual differences (e.g., work values, goals, and needs) to influence relevant employees’ outcomes. As work values and HRM values are likely to be deeply anchored within the individual, it would be also valuable to examine the specific and complementary nature of both types of values. However, by developing a comprehensive measure of HRM values, we hope to provide a psychometric anchor upon which to build these future investigations. Indeed, in a field where most studies have to rely on a diverse set of unvalidated measures to assess HRM practices due to a lack of validated instruments (Boon et al., 2019), we hope to provide a more psychometrically rigorous alternative to researchers. As our measure of HRM values is closely connected to an already validated measure of HRM practices (Geringer et al., 2002; Fabi et al., 2015), researchers can now benefit from two complementary tools upon which to build their investigations.

Beyond these more theoretical and practical implications, the current research also has methodological implications. Indeed, sound psychometric measurement goes hand in hand with latent variable models (Marsh and Hau, 2007). Latent variable models, rather than simply being a tool allowing researchers to study the psychometric properties of their measures, provide a way to assess more complex chains of relations in a way that incorporate a correction for unreliability (i.e., random measurement error). Whereas our results showed that the reliability of scores obtained on each of our factors was generally acceptable to satisfactory, some factors remained associated with reliability coefficients located at the lower bound of acceptability (selection) or under that lower bound (work-life balance, selection). On the one hand, these results indicate that practitioners should be cautious when using these specific subscales, at least pending further investigations of their psychometric properties. On the other hand, these results also further highlight the importance of latent variable models for research seeking to investigate the role played by these practices. Critically, tests of interactions effects, such as those that would be needed to assess how HRM values may modify the impact of HRM practices, are notably sensitive to unreliability when tested using manifest variables (i.e., the sum or average of the items forming a scale), but remain unaffected by unreliability when tested using latent variable models (e.g., Marsh et al., 2013).

In addition, the present study also provides one further illustration of the utility of ESEM for researchers seeking to better understand the psychometric properties of scores obtained on their measures, as well as to develop shorter forms of longer instruments. Indeed, whereas the CFA solution obtained for the original HRM-VS failed to achieve a proper level of fit to data, the parameter estimates obtained as part of this solution proved mostly (i.e., as all factors seemed to be defined properly by their *a priori* indicators) useless to guide the development of a more psychometrically sound (and shorter) version. In contrast, the ESEM solution made it relatively easy to identify problematic items, thus adding to previous demonstrations of the value of this novel statistical approach (e.g., Levesque-Côté et al., 2018). Importantly, even with psychometrically sound measures, such as our final, short, version the HRM-VS, statistical research has also shown that ESEM tends to result in more accurate estimate of latent factors, and in more accurate estimates of factor correlations (Asparouhov et al., 2015). This last observation is particularly important as it suggest that, rather than simply providing a novel, and perhaps more elegant, way of assessing the factor structure of scores obtained on a measurement instrument, ESEM also increases our ability to obtain more accurate estimates of predictions (Mai et al., 2018). Indeed, by reducing the correlations among factors, ESEM reduces the risk that predictive results will be tainted by multicollinearity.

### 4.2. Limitations

Although our results support the proposed conceptualization of HRM values and provide strong evidence for the construct validity (factor structure, reliability, and criterion-related validity) of scores obtained on the HRM-VS, certain limitations should be considered. First, although we were able to identify a relevant set of items allowing us to assess the value ascribed by employees to nine distinct HRM practices, the two items used to measure induction were found to be problematic. As induction practices are typically tied in the socialization process whereby the organization welcomes new employees, future studies should aim at improving the item content of this dimension at different stages of the organization life and of employees’ socialization. Second, this study relied exclusively on self-report measures, which increases the risk of bias in responses. Future studies should thus consider adopting more objective outcome measures (e.g., job performance, absenteeism, turnover) to obtain a more accurate assessment of the criterion-validity of scores obtained on this measure. Third, the present study relied on a cross-sectional design, which does not allow us to establish the directionality of the associations tested in relation to the criterion-related validity of scores obtained on our measure. Fourth, this study relied on a fairly limited set of outcomes. While the relations between several HRM values and intrinsic and extrinsic job satisfaction has been established, further research should enhance our understanding of behavioral (e.g., task performance, organizational citizenship behaviors) and other attitudinal (e.g., organizational commitment) outcomes. Further studies are also needed to improve our understanding of the predictors of HRM values. For example, would the presence (or the absence) of particular HRM practices affect the core HRM values. Fifth, our sample is limited to French-Canadian employees working in the service, manufacturing or public sectors. With respect to the generalization, our results should thus be replicated among more diversified samples of employees from other cultures and types of industries.

### 4.3. Practical implications

This HRM-VS holds practical implications for both researchers and organizations. This new concise but complete measure could help better guide organizations in their strategic human resource management. By its specific nature, the HRM-VS opens way to evaluate what employees really want out of their organizations’ HRM systems. When rethinking their HR policies, organizations using this scale would thus benefit from an enhanced understanding about the most beneficial practices to focus on, considering their employees’ values, instead of relying on a one-size-fits-all approach of “best practices.” From a motivation standpoint, HRM managers still frequently focus either on punitive measures or incentives to regulate employee motivation and performance (Manganelli et al., 2018). To deliver a new strategic HRM approach, it may be valuable for managers to focus on creating opportunities to express or encourage employee HRM values at work, through the best fit in tailoring strategies and practices.

## Data availability statement

The data analyzed in this study is subject to the following licenses/restrictions: The raw data supporting the conclusions of this article will be made available by the authors, without undue reservation. Requests to access these datasets should be directed to bruno.fabi@uqtr.ca.

## Ethics statement

The studies involving human participants were reviewed and approved by Comité d’éthique de la recherche avec des êtres humains de l’Université du Québec à Trois-Rivières. The patients/participants provided their written informed consent to participate in this study.

## Author contributions

SD-R: conception of the study under the supervision of CF and SA, data collection under the supervision of BF, analysis and interpretation of the data under the supervision of AM. AM, CF, SA, and BF: critical review of the manuscript. All authors contributed to the article and approved the submitted version.

## Funding

Preparation of this manuscript was supported by grants from the Fonds de Recherche du Québec – Société et Culture (2019-SE1-252542) and the Social Science and Humanity Research Council of Canada (435-2018-0368).

## Conflict of interest

The authors declare that the research was conducted in the absence of any commercial or financial relationships that could be construed as a potential conflict of interest.

## Publisher’s note

All claims expressed in this article are solely those of the authors and do not necessarily represent those of their affiliated organizations, or those of the publisher, the editors and the reviewers. Any product that may be evaluated in this article, or claim that may be made by its manufacturer, is not guaranteed or endorsed by the publisher.
