# Supplementary material for: Employee human resource management values: validation of a new concept and scale
Source: Front Psychol. 2023 May 5;14:1049657. doi: 10.3389/fpsyg.2023.1049657 (PMC10196065; doi:10.3389/fpsyg.2023.1049657)
Supplement: Supplementary file 1 [file Data_Sheet_1.docx]

Supplementary Material

**Table S1**

Standardized factor loadings (λ) and item uniquenesses ($\delta$) for the exploratory structural equation modeling (ESEM) solution of the long version.

|  | F1 (λ) | F2 (λ) | F3 (λ) | F4 (λ) | F5 (λ) | F6 (λ) | F7 (λ) | F8 (λ) | F9 (λ) | F10 (λ) | $\delta$ |
| --- | --- | --- | --- | --- | --- | --- | --- | --- | --- | --- | --- |
| B1AA | .515** |  |  |  |  |  |  |  |  |  | .735** |
| B1BB | .421** |  |  |  |  |  |  |  |  |  | .823** |
| B1CC | .411** |  |  |  |  |  |  |  |  |  | .831** |
| B1DD | .507** |  |  |  |  |  |  |  |  |  | .743** |
| B1EE | .577** |  |  |  |  |  |  |  |  |  | .667** |
| B1FF | .513** |  |  |  |  |  |  |  |  |  | .737** |
| B2AA |  | .452** |  |  |  |  |  |  |  |  | .795** |
| B2BB |  | .390** |  |  |  |  |  |  |  |  | .848** |
| B2CC |  | .702** |  |  |  |  |  |  |  |  | .507** |
| B2DD |  | .739** |  |  |  |  |  |  |  |  | .454** |
| B2EE |  | .640** |  |  |  |  |  |  |  |  | .590** |
| B2FF |  | .545** |  |  |  |  |  |  |  |  | .703** |
| B2GG |  | .689** |  |  |  |  |  |  |  |  | .526** |
| B2HH |  | .596** |  |  |  |  |  |  |  |  | .645** |
| B3AA |  |  | .452** |  |  |  |  |  |  |  | .810** |
| B3BB |  |  | .668** |  |  |  |  |  |  |  | .554** |
| B3CC |  |  | .683** |  |  |  |  |  |  |  | .533** |
| B3DD |  |  | .661** |  |  |  |  |  |  |  | .563** |
| B3EE |  |  | .665** |  |  |  |  |  |  |  | .558** |
| B3FF |  |  | .610** |  |  |  |  |  |  |  | .627** |
| B3GG |  |  | .606** |  |  |  |  |  |  |  | .633** |
| B4AA |  |  |  | .520** |  |  |  |  |  |  | .730** |
| B4BB |  |  |  | .703** |  |  |  |  |  |  | .506** |
| B4CC |  |  |  | .760** |  |  |  |  |  |  | .422** |
| B4DD |  |  |  | .810** |  |  |  |  |  |  | .343** |
| B4EE |  |  |  | .799** |  |  |  |  |  |  | .361** |
| B4FF |  |  |  | .797** |  |  |  |  |  |  | .364** |
| B4GG |  |  |  | .699** |  |  |  |  |  |  | .511** |
| B4HH |  |  |  | .648** |  |  |  |  |  |  | .580** |
| B5AA |  |  |  |  | .599** |  |  |  |  |  | .641** |
| B5BB |  |  |  |  | .551** |  |  |  |  |  | .697** |
| B5CC |  |  |  |  | .505** |  |  |  |  |  | .745** |
| B5DD |  |  |  |  | .552** |  |  |  |  |  | .695** |
| B5EE |  |  |  |  | .491** |  |  |  |  |  | .759** |
| B5FF |  |  |  |  | .481** |  |  |  |  |  | .768** |
| B5GG |  |  |  |  | .466** |  |  |  |  |  | .783** |
| B5HH |  |  |  |  | .461** |  |  |  |  |  | .787** |
| B6AA |  |  |  |  |  | .765** |  |  |  |  | .415** |
| B6BB |  |  |  |  |  | .488** |  |  |  |  | .762** |
| B7AA |  |  |  |  |  |  | .683** |  |  |  | .533** |
| B7BB |  |  |  |  |  |  | .647** |  |  |  | .581** |
| B7CC |  |  |  |  |  |  | .695** |  |  |  | .516** |
| B7DD |  |  |  |  |  |  | .665** |  |  |  | .557** |
| B7EE |  |  |  |  |  |  | .647** |  |  |  | .581** |
| B7FF |  |  |  |  |  |  | .721** |  |  |  | .480** |
| B7GG |  |  |  |  |  |  | .645** |  |  |  | .585** |
| B7HH |  |  |  |  |  |  | .717** |  |  |  | .486** |
| B7II |  |  |  |  |  |  | .649** |  |  |  | .579** |
| B8AA |  |  |  |  |  |  |  | .563** |  |  | .683** |
| B8BB |  |  |  |  |  |  |  | .611** |  |  | .627** |
| B8CC |  |  |  |  |  |  |  | .440** |  |  | .806** |
| B8DD |  |  |  |  |  |  |  | .703** |  |  | .506** |
| B8EE |  |  |  |  |  |  |  | .699** |  |  | .511** |
| B8FF |  |  |  |  |  |  |  | .344** |  |  | .881** |
| B8GG |  |  |  |  |  |  |  | .351** |  |  | .877** |
| B9AA |  |  |  |  |  |  |  |  | .627** |  | .607** |
| B9BB |  |  |  |  |  |  |  |  | .744** |  | .446** |
| B9CC |  |  |  |  |  |  |  |  | .730** |  | .467** |
| B9DD |  |  |  |  |  |  |  |  | .680** |  | .538** |
| B9EE |  |  |  |  |  |  |  |  | .170** |  | .971** |
| B9FF |  |  |  |  |  |  |  |  | .597** |  | .643** |
| B9GG |  |  |  |  |  |  |  |  | .777** |  | .397** |
| B9HH |  |  |  |  |  |  |  |  | .726** |  | .473** |
| B10AA |  |  |  |  |  |  |  |  |  | .574** | .671** |
| B10BB |  |  |  |  |  |  |  |  |  | .671** | .550** |
| B10CC |  |  |  |  |  |  |  |  |  | .747** | .442** |
| B10DD |  |  |  |  |  |  |  |  |  | .730** | .467** |
| B10EE |  |  |  |  |  |  |  |  |  | .704** | .505** |
| B10FF |  |  |  |  |  |  |  |  |  | .491** | .759** |
| B10GG |  |  |  |  |  |  |  |  |  | .611** | .627** |
| B10HH |  |  |  |  |  |  |  |  |  | .511** | .739** |
| B10II |  |  |  |  |  |  |  |  |  | .421** | .822** |
| B10JJ |  |  |  |  |  |  |  |  |  | .437** | .809** |

*Note.* **p* < .05; ***p* < .01;  F1 = Work-life balance; F2 = Leadership; F3 = Communication and participation; F4 = Performance appraisal; F5 = Selection; F6 = Induction; F7 = Training and development; F8 = Compensation; F9 = Benefits; F10 = Work design. B1 = Work-life balance items; B2 = Leadership items; B3 = Communication and participation items; B4 = Performance appraisal items; B5 = Selection items; B6 = Induction items; B7 = Training and development items; B8 = Compensation items; B9 = Benefits items; B10= Work design items. λ = standardized factor loading; δ = standardized item uniquenesses.

**Table S2**

Factor correlations from the confirmatory factor analytic (CFA; under the diagonal) and exploratory structural equation modeling (ESEM; above the diagonal) solution of the long version.

|  | F1 | F2 | F3 | F4 | F5 | F6 | F7 | F8 | F9 | F10 |
| --- | --- | --- | --- | --- | --- | --- | --- | --- | --- | --- |
| F1 | - | .140 | .171* | .151* | .225* | .057 | .229** | .201** | .272** | .090 |
| F2 | .310** | - | .490** | .478** | .247* | .127 | .440** | .178** | .308** | .451** |
| F3 | .237** | .728** | - | .540** | .336* | .053 | .432** | .133* | .332** | .376* |
| F4 | .231** | .588** | .689** | - | .380* | -.017 | .448** | .296** | .217** | .364** |
| F5 | .297** | .558** | .627** | .605** | - | .109* | .438** | .123 | .332* | .202 |
| F6 | .274** | .533** | .645** | .515** | .582** | - | .105* | -.084* | .147* | -.012 |
| F7 | .307** | .601** | .608** | .553** | .648** | .717** | - | .136** | .455** | .410* |
| F8 | .341** | .412** | .335** | .494** | .449** | .309** | .441** | - | .063 | .064 |
| F9 | .431** | .446** | .516** | .350** | .520** | .596** | .599** | .343** | - | .341** |
| F10 | .333** | .684** | .711** | .585** | .654** | .612** | .714** | .437** | .607** | - |

*Note.* **p* < .05; ***p* < .01; F1 = Work-life balance; F2 = Leadership; F3 = Communication and participation; F4 = Performance appraisal; F5 = Selection; F6 = Induction; F7 = Training and development; F8 = Compensation; F9 = Benefits; F10 = Work design.

**Table S3**

Standardized factor loadings (λ) and item uniquenesses ($\delta$) from the confirmatory factor analytic solution of the long version.

|  | F1 (λ) | F2 (λ) | F3 (λ) | F4 (λ) | F5 (λ) | F6 (λ) | F7 (λ) | F8 (λ) | F9 (λ) | F10 (λ) | $\delta$ |
| --- | --- | --- | --- | --- | --- | --- | --- | --- | --- | --- | --- |
| B1AA | **.424**** | .053 | -.014 | .089 | -.133 | -.087 | -.061 | .029 | .022 | .186* | .749** |
| B1BB | **.549**** | .133* | -.098 | .034 | -.018 | .128* | .006 | .013 | -.171** | .035 | .677** |
| B1CC | **.554**** | -.051 | -.039 | -.034 | .038 | .054 | .079 | -.032 | -.082* | -.007 | .696** |
| B1DD | **.372**** | -.027 | .086 | .031 | -.085 | -.250** | -.037 | .114* | .077 | .075 | .736** |
| B1EE | **.459**** | .067 | -.070 | -.029 | -.019 | .021 | -.046 | -.029 | .213** | .056 | .705** |
| B1FF | **.394**** | .086 | .056 | -.017 | -.072 | -.065 | .002 | .021 | .207** | -.105 | .752** |
| B2AA | .090 | **.251** | .105 | .149 | .053 | -.064 | .151* | .102 | -.008 | -.191** | .720** |
| B2BB | .052 | **.166** | .105 | .144 | .148 | -.133 | .123 | .193** | -.069 | -.157* | .715** |
| B2CC | .056 | **.721**** | -.031 | -.062 | .013 | .006 | .030 | -.026 | -.009 | .085 | .451** |
| B2DD | .027 | **.837**** | -.010 | .015 | -.051 | -.043 | -.045 | -.011 | .044 | -.040 | .354** |
| B2EE | .065 | **.491**** | .187* | .026 | .052 | -.025 | .022 | .113* | -.030 | -.075 | .574** |
| B2FF | .072 | **.454**** | .136 | .001 | .157* | .057 | .020 | -.070 | -.045 | -.089 | .668** |
| B2GG | -.035 | **.621**** | -.004 | .000 | -.025 | -.006 | .084 | -.034 | .014 | .112 | .498** |
| B2HH | -.048 | **.409**** | .162 | .002 | .071 | -.063 | -.022 | -.093* | .009 | .192* | .613** |
| B3AA | .104 | -.045 | **.367*** | -.005 | .173 | -.065 | .066 | -.033 | .105* | -.076 | .727** |
| B3BB | -.029 | .229** | **.463**** | .174* | -.030 | -.021 | -.005 | -.032 | .014 | -.073 | .546** |
| B3CC | -.102* | .109 | **.472*** | .112 | .001 | -.018 | .097 | -.043 | .054 | .023 | .548** |
| B3DD | -.102* | .042 | **.547**** | .091 | -.023 | .120* | .110 | -.005 | -.007 | .017 | .534** |
| B3EE | .014 | .082 | **.590**** | -.002 | -.025 | -.034 | -.060 | .000 | .031 | .165 | .518** |
| B3FF | -.013 | .101 | **.465*** | .041 | -.040 | -.138 | -.103 | -.003 | .004 | .323** | .519** |
| B3GG | .054 | .016 | **.476**** | .064 | .126* | .072 | .036 | -.093* | .077 | -.011 | .585** |
| B4AA | .016 | .004 | .179* | **.329**** | .075 | -.018 | -.097 | .433** | -.10* | -.052 | .537** |
| B4BB | -.015 | -.031 | .021 | **.656**** | .105 | -.024 | .018 | .114* | .002 | -.067 | .471** |
| B4CC | -.017 | .117* | .013 | **.692**** | -.061 | .056 | -.062 | .009 | .037 | .095 | .412** |
| B4DD | .023 | -.001 | .018 | **.806**** | -.036 | -.038 | .084* | -.125 ** | -.009 | .027 | .315** |
| B4EE | -.021 | -.016 | .048 | **.817**** | .019 | -.040 | .051 | -.051 | -.015 | -.083 | .326** |
| B4FF | .002 | -.037 | -.068 | **.887**** | .012 | .012 | -.007 | -.080* | .021 | .012 | .319** |
| B4GG | .066 | .047 | .125 | **.554**** | -.040 | .071 | -.014 | -.055 | .040 | .132* | .487** |
| B4HH | .000 | -.003 | .076 | **.519**** | .022 | .010 | -.013 | .191** | .032 | .016 | .563** |
| B5AA | -.017 | .057 | .086 | .156* | **.220*** | -.023 | .073 | -.015 | .064 | .176* | .682** |
| B5BB | -.039 | .159* | .022 | .125* | **.284*** | .002 | -.031 | .085 | .025 | .074 | .758** |
| B5CC | -.010 | .094 | .038 | .000 | **.389*** | -.024 | .072 | -.006 | -.090 | .134 | .749** |
| B5DD | -.061 | .032 | .038 | -.022 | **.551*** | -.014 | .097 | .024 | .014 | -.023 | .633** |
| B5EE | -.041 | .055 | -.051 | .011 | **.497**** | -.106 | .039 | .064 | .140* | -.060 | .679** |
| B5FF | -.045 | .044 | .020 | -.007 | **.532*** | -.024 | -.134* | .102 | .015 | .122 | .698** |
| B5GG | -.023 | -.031 | .045 | .103 | **.280*** | .071 | .074 | -.017 | -.048 | .174* | .792** |
| B5HH | .056 | -.007 | .104 | .032 | **.297*** | .020 | -.015 | .045 | .054 | .085 | .805** |
| B6AA | -.020 | .051 | .169* | .059 | .052 | **.037** | .327** | -.085 | .133* | .042 | .629** |
| B6BB | .175** | .023 | .034 | .121* | .094 | **-.042** | .164 * | -.014 | .107* | -.071 | .804** |
| B7AA | .009 | .091 | -.067 | .125* | .098 | -.132 | **.433**** | -.031 | .101* | .134 | .521** |
| B7BB | .160* | -.006 | .005 | .030 | .197* | -.178 | **.487**** | -.071 | .058 | .021 | .507** |
| B7CC | -.020 | .084 | -.070 | .149* | .140* | -.115 | **.556**** | .096* | -.058 | .019 | .473** |
| B7DD | .093 | .016 | -.022 | .016 | .126 | -.103 | **.616**** | -.076* | -.100* | .095 | .509** |
| B7EE | -.035 | .118 | .051 | .006 | -.168* | .135* | **.641**** | .058 | .024 | -.020 | .506** |
| B7FF | -.067 | -.031 | .109* | -.104* | -.105 | .145* | **.759**** | .097* | .034 | .061 | .383** |
| B7GG | -.040 | .022 | .068 | -.060 | -.016 | .074 | **.673**** | .113* | .027 | -.086 | .527** |
| B7HH | -.002 | -.011 | .017 | -.008 | -.039 | -.032 | **.663**** | -.011 | .039 | .128* | .478** |
| B7II | -.035 | .009 | -.025 | .072 | -.058 | -.016 | **.560**** | -.012 | .127* | .071 | .572** |
| B8AA | .110* | .007 | .122 | -.115* | .040 | -.005 | .061 | **.557**** | .112* | -.044 | .598** |
| B8BB | -.126* | -.068 | .024 | .040 | .044 | -.034 | .022 | **.652**** | -.006 | -.081 | .500** |
| B8CC | .088 | .015 | -.095 | .134* | .132 | .219** | -.005 | **.267**** | .174** | -.004 | .731** |
| B8DD | -.062 | .045 | -.079 | .114* | .034 | -.043 | .002 | **.745**** | -.092* | .108* | .364** |
| B8EE | -.046 | -.003 | -.115 | .155* | .027 | -.049 | .133* | **.531**** | .053 | .160** | .549** |
| B8FF | -.025 | .133* | -.042 | .033 | .150 | .227* | .021 | **.124*** | .193** | .104 | .728** |
| B8GG | -.034 | .200* | -.016 | -.046 | -.019 | .181 | .191* | **.157*** | .093 | .155* | .720** |
| B9AA | .121* | .033 | .084 | -.029 | -.050 | .056 | .021 | .303** | **.491**** | .024 | .527** |
| B9BB | -.143** | .085 | -.035 | .015 | .072 | -.037 | -.020 | .039 | **.792**** | -.025 | .372** |
| B9CC | .040 | .068 | -.020 | .114* | -.076 | .137 * | .007 | -.012 | **.667**** | .049 | .422** |
| B9DD | .111* | -.057 | .016 | .013 | .151 | .002 | .146* | -.147** | **.532**** | -.010 | .493** |
| B9EE | .283** | .107 | -.001 | .036 | .013 | .190* | .038 | .212** | **-.089** | .008 | .774** |
| B9FF | .113* | -.062 | .097 | .078 | -.065 | -.084 | .127* | .122* | **.406**** | .127 | .591** |
| B9GG | -.063 | .011 | .083* | -.046 | -.034 | -.131* | .011 | .025 | **.819**** | .025 | .321** |
| B9HH | .158** | -.059 | .037 | -.014 | .092 | -.022 | .008 | -.078 | **.650**** | .037 | .439** |
| B10AA | .058 | -.107* | .130* | .036 | .105 | -.140 | -.004 | .006 | .102* | **.495**** | .600** |
| B10BB | .059 | -.025 | .150 | .009 | .037 | -.138 | .008 | .078* | .027 | **.627**** | .439** |
| B10CC | .012 | .109 | .037 | .022 | .029 | -.029 | .117* | .073 | .007 | **.584**** | .429** |
| B10DD | .020 | .163* | -.057 | .023 | .003 | -.091 | .100 | .067 | .037 | **.616**** | .401** |
| B10EE | -.045 | .063 | .004 | .085 | .029 | -.126 | .149* | .065 | .033 | **.539**** | .466** |
| B10FF | .104 | .061 | .093 | .021 | .181 | .288* | .069 | -.030 | .048 | **.166** | .663** |
| B10GG | .051 | -.001 | .177* | .108* | .042 | .325** | .069 | -.060 | -.011 | **.389** | .522** |
| B10HH | .022 | .028 | .133 | .105 | .107 | .381** | .071 | -.051 | .084 | **.191** | .576** |
| B10II | .153* | -.054 | .111 | -.044 | .196 | .105 | .192* | -.036 | -.038 | **.167** | .758** |
| B10JJ | .001 | .085 | -.016 | -.036 | .196* | .350** | .024 | -.030 | .315** | **.117** | .563** |

*Note.* **p* < .05; ***p* < .01; Main factor loadings are indicated in bold; F1 = Work-life balance; F2 = Leadership; F3 = Communication and participation; F4 = Performance appraisal; F5 = Selection; F6 = Induction; F7 = Training and development; F8 = Compensation; F9 = Benefits; F10 = Work design. B1 = Work-life balance items; B2 = Leadership items; B3 = Communication and participation items; B4 = Performance appraisal items; B5 = Selection items; B6 = Induction items; B7 = Training and development items; B8 = Compensation items; B9 = Benefits items; B10= Work design items. λ = standardized factor loading; δ = standardized item uniquenesses.

**Table S4**

Latent Correlations

| Variable | 1 | 2 | 3 | 4 | 5 | 6 | 7 | 8 | 9 | 10 | 11 | 12 |
| --- | --- | --- | --- | --- | --- | --- | --- | --- | --- | --- | --- | --- |
| 1. Gender (0 = female, 1 = male) | - |  |  |  |  |  |  |  |  |  |  |  |
| 2. Work-life balance | -.195* | - |  |  |  |  |  |  |  |  |  |  |
| 3. Leadership | -.167** | .211* | - |  |  |  |  |  |  |  |  |  |
| 4. Communication and participation | -.032 | .232** | .445** | - |  |  |  |  |  |  |  |  |
| 5. Performance appraisal | -.177** | .186* | .419** | .476** | - |  |  |  |  |  |  |  |
| 6. Selection | .004 | .143* | .225** | .305** | .341** | - |  |  |  |  |  |  |
| 7. Training and development | -.127* | .192* | .439** | .359** | .422** | .369** | - |  |  |  |  |  |
| 8. Compensation | .032 | .309** | .198** | .194** | .310** | .279** | .246** | - |  |  |  |  |
| 9. Benefits | -.122* | .278** | .258** | .324** | .198** | .333** | .431** | .127* | - |  |  |  |
| 10. Work design | -.137* | .208* | .319** | .437** | .337** | .234** | .374** | .212** | .300** | - |  |  |
| 11. Intrinsic job satisfaction | -.011 | -.046 | .024 | .235** | .162** | .243** | .075 | .018 | .079 | .235** | - |  |
| 12. Extrinsic job satisfaction | -.018* | -.090 | .173** | -.004 | .193** | .169* | .086 | .003 | -.051 | .083 | .689** | - |

*Note.* **p* < .05; ***p* < .01; All latent variables have a mean of 0 and a SD of 1.
